# Individual prediction of hemispheric similarity of functional connectivity during normal aging

**DOI:** 10.3389/fpsyt.2022.1016807

**Published:** 2022-09-26

**Authors:** Yingteng Zhang

**Affiliations:** Department of Mathematics, Taizhou University, Jiangsu Province, Taizhou, China

**Keywords:** hemispheric similarity of functional connectivity, functional MRI, normal aging, individual recognition, global signal

## Abstract

In the aging process of normal people, the functional activity pattern of brain is in constant change, and the change of brain runs through the whole life cycle, which plays a crucial role in the track of individual development. In recent years, some studies had been carried out on the brain functional activity pattern during individual aging process from different perspectives, which provided an opportunity for the problem we want to study. In this study, we used the resting-state functional magnetic resonance imaging (rs-fMRI) data from Cambridge Center for Aging and Neuroscience (Cam-CAN) database with large sample and long lifespan, and computed the functional connectivity (FC) values for each individual. Based on these values, the hemispheric similarity of functional connectivity (HSFC) obtained by Pearson correlation was used as the starting point of this study. We evaluated the ability of individual recognition of HSFC in the process of aging, as well as the variation trend with aging process. The results showed that HSFC could be used to identify individuals effectively, and it could reflect the change rule in the process of aging. In addition, we observed a series of results at the sub-module level and find that the recognition rate in the sub-module was different from each other, as well as the trend with age. Finally, as a validation, we repeated the main results by human brainnetome atlas (BNA) template and without global signal regression, found that had a good robustness. This also provides a new clue to hemispherical change patterns during normal aging.

## Introduction

In recent years, a large number of studies (1–3) have used the combination of pattern recognition and brain image data to distinguish healthy elderly people from Alzheimer’s disease (AD) patients, and achieve good results. In addition to using structural MRI (sMRI) data to explore cortical atrophy and white matter fiber tracts abnormalities in specific areas of AD, several studies (4, 5) have also used fMRI data to explore differences in brain functional activity between healthy elderly people and AD patients. The above researches reflect the distribution pattern of brain structure and function in people with abnormal aging (i.e., suffering from common nervous system diseases such as AD). However, in the life cycle of normal people, from youth, middle age to old age, the pattern of brain functional activity is constantly changing. There is a lack of relevant research on the change rule with the aging process, which has always been the focus of attention in the field of cognitive neuroscience.

To investigate the difference pattern of individual brain functional activity during normal aging, some scholars (6–8) study a series of metrics derived from fMRI, such as regional homogeneity (ReHo), amplitude of low frequency fluctuation (ALFF) and functional connectivity (FC). Most of these indicators are studied at the whole brain level, some specific regions of interest (ROI) or homologous brain regions, and the correlation of activity patterns between the left and right hemispheres is not clear. In recent years, pattern recognition has been applied more and more widely in neuroimaging and numerous individual recognition methods are constantly innovating. For example, Finn et al. (9) used the rs-fMRI and task fMRI (tfMRI) data of a large sample from human Connectome Project (HCP) in 2015. Their research demonstrated that functional connectivity, as a kind of “fingerprinting,” could effectively identify individuals from large samples, and that the sub-network with the most significant difference among individuals could well predict individual differences in fluid intelligence. In addition, Kaufmann et al. (10) used this fingerprinting method in 2017 to show that delayed brain network development during adolescence was associated with decreased mental health. However, the effectiveness of this “fingerprinting” approach in identifying individuals during normal aging remains unclear.

Brain changes occur throughout the life cycle and play a critical role in individual developmental trajectories for cognition, social functioning, adaptability, personality and mental health. Due to the great potential of neuroplasticity and the continuous development of environmental sensitivity, some scholars hypothesize that functional connectivity shapes individual differences in individual maturation and aging mechanisms. In recent years, several studies (11–13) have made use of Cam-CAN database to study the brain functional activity pattern of individual aging process from different perspectives, which provide an opportunity for our research.

Here, we proposed the metric of the left and right hemispheric similarity of functional connectivity (HSFC) to explore whether the hemispheric similarity had the characteristics of individual differences in groups of different ages and how it changed during aging. In particular, we used the Cam-CAN dataset for a population aged 18–88 years and constructed hemispheric functional connectivity networks for rs-fMRI data of each individual. Then, the HSFC computed by Pearson correlation was used as the starting point of this study to evaluate the individual identification ability of HSFC in the aging process and its correlation with age. In addition, we observed a series of results of HSFC at the sub-module level. Finally, as a validation, we repeated the main results through another functional template and no global signal regression (NGSR).

## Materials and methods

### Subjects

The Cam-CAN Stage 2 dataset^1^ (14) included 646 subjects with T1 and rs-fMRI data (age range: 18∼88 years, 314 males) was used. All the subjects were native English speakers, had normal or corrected vision and hearing, scored 25 or above on the mini-mental state examination (MMSE), and had no neurological disorders. It was worth noting that 4 subjects are excluded from this dataset due to incomplete data collection. Thus, a total of 642 subjects entered the preprocessing step. Ethical approval was approved by the University of Cambridge’s Research Ethics Committee. All subjects gave written informed consent.

All scans were performed using the standard 3T Tim Trio (Siemens) with 32 channel coils. The rs-fMRI scans were obtained using EPI sequences: whole brain coverage; 261 volumes, each volume contains 32 axial slices; layer thickness 3.7 mm with an 20% inter-slice gap; TR = 1,970 ms; TE = 30 ms; FOV = 192 × 192 mm^2^; flip angle = 78°; voxel size = 3 × 3 × 4.44 mm^3^. High resolution T1-weighted structure images were obtained using MPRAGE sequence, and the parameters were as follows: TR = 2,250 ms; TE = 2.99 ms; TI = 900 ms; FOV = 256 × 240 × 192 mm^3^; flip angle = 9°; voxel size = 1 mm; isotropy; generalized automatic calibration partial parallel acquisition (GRAPPA) acceleration factor = 2.

### Data processing

Firstly, using the FUGUE tool of the FSL package to accomplish the fieldmap correction.^2^ According to the phase difference image and short TE amplitude images to get rad images and then used the rad images of EPI image correction. Then DPABI toolbox was used to preprocess the resultant rs-fMRI images (15), including the following steps: ➀ removed the first 10 time points; ➁ time layer correction; ➂ head movement correction; ➃ the diffeomorphic anatomical registrations through exponentiated lie algebra (DARTEL) (16) segmentation method dealt with sMRI scans and used it to normalize rs-fMRI scans. ➄ standardization; ➅ regression covariables (including Friston’s 24 head movement parameters (17), global signal, average signal of white matter and cerebrospinal fluid); ➆ bandpass filtering (0.01–0.1 Hz). It was worth noting that there was some controversy over whether the global signal should be regressed during rs-fMRI data preprocessing (18–20). In 2009, there existed opposite recommendations about whether GSR should be used in the processing of rs-fMRI data (18, 19). Murphy et al. was the first to show that GSR mathematically mandates the presence of anti-correlations (19). Because anti-correlations following GSR could be an artifact of the processing technique, Murphy et al. concluded that GSR should not be used. However, Fox et al. found that several characteristics of anti-correlated networks could not be attributed to GSR. Because GSR enhanced the detection of system-specific correlations and improved the correspondence between resting-state correlations and anatomy, they concluded that GSR can be beneficial (18). Therefore, we also calculated NGSR in the step of regression covariate to explore the influence of global signal on the results. In the preprocess, 14 subjects with a head movement of more than 3 mm and 3° and 1 subject with segmentation failure were removed. A total of 627 subjects were included in the analysis. There were 166 subjects in the Young group (18∼39 years old), 197 subjects in the Middle group (40∼59 years old), and 264 subjects in the Old group (60∼88 years old). The information of subjects was shown in Table 1. There was no significant difference in gender (*P* = 0.871) and significant difference in age (*P* < 0.0001). The statistical analysis of basic information was obtained through SPSS22.0.

**TABLE 1:** Subject demographics.

	Young	Middle	Old	*P*-value
Sample size	166	197	264	
Gender (male/female)	79/87	95/102	132/132	0.871
Age (years)	30.56 ± 5.68	49.21 ± 5.67	72.71 ± 7.53	<0.0001

The ages are shown as mean ± standard deviation (SD). Columns on the right display P-value by F-test for age and the gender computes P-value by chi-square test.

### Constructing functional network

**FIGURE 1:**
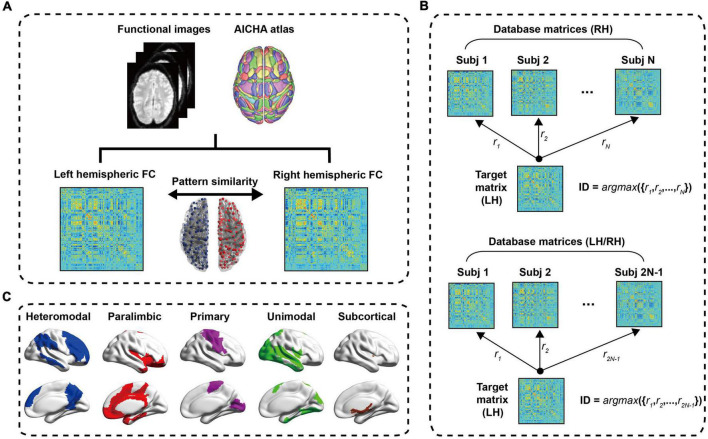
The process flow chart. **(A)** Data preprocessing. After a series of preprocessing steps, a 384 × 384 symmetric resting state FC matrix is obtained, and the left hemisphere and right hemisphere is 192 × 192 symmetric connection matrices, respectively. The Pearson correlation of left hemisphere and right hemisphere of intra-subject is defined as the hemispheric similarity of functional connectivity (HSFC). **(B)** Schematic diagram of individual identification; **(C)** the cortical distribution of the five sub-modules. LH, Left hemisphere; RH, Right hemisphere.

The construction process of functional network was shown in Figure 1A. For Cam-CAN data, we used the atlas of intrinsic connectivity of homotopic areas (AICHA) (21) to extract the average time series of each ROI. The atlas divided the brain into 384 regions (192 regions in each hemisphere), containing 344 cortical regions and 40 subcortical regions. It had been used in some studies to divide the brain for FC and brain network analysis (22–24). For each subject, we obtained the mean time series of 384 regions through the time series of all voxels in each ROI. The FC between two brain regions was obtained by calculating Pearson correlation coefficients of average time series. Finally, each subject obtained a 384 × 384 symmetric FC matrix. Each intra-hemisphere network was a 192 × 192 symmetric FC matrix and had been used Fisher-z transform to make the statistical normalization.

In order to explore the contribution of different ROI to individual recognition, we further subdivided the hemispheric functional network into five sub-modules (i.e., heteromodal, paralimbic, primary, unimodal and subcortical) based on functional hierarchy (25). This functional hierarchy was based on studies of anatomy, electrophysiology, behavior, injury, and functional imaging in non-human primates and humans. The heteromodal and unimodal areas were most closely involved in perceptual elaboration and motor planning. The paralimbic areas played a critical role in channeling emotion and motivation to behaviorally relevant intrapsychic and extrapersonal targets. The primary included primary sensory cortex, primary motor cortex, primary visual cortex, primary auditory cortex, primary somatosensory cortex and primary gustatory cortex and these cortices mainly responsible for the control of motor, visual processing, auditory processing and other functions. The subcortical included insula, amygdala, putamen and thalamus. Among them, the thalamus relays communication among subcortical and cortical regions and played a central role in the integration of sensory information. The cortical distribution of the five sub-modules was shown in Figure 1C. Many studies had used these sub-modules (26–28).

### Individual identification steps for hemispherical functional networks

The individual identification method used in this paper was a reference to the work of Finn et al. (9). Finn et al. used the rs-fMRI and tfMRI data from HCP database and this research demonstrated that functional connectivity, as a kind of “fingerprinting,” could effectively identify individuals from large samples. The difference between the individual recognition of Finn et al. and ours was that Finn et al. computed the Pearson correlation between the functional connectivity of the whole brain of an individual and the functional connectivity of the whole brain of another scan, while we computed the Pearson correlation between the functional connectivity of the left and right intra-hemispheres of an individual to complete the recognition process. Figure 1B showed the process of the LH recognizing the RH in individual. First, created database matrices containing right hemisphere FC matrices for all subjects. *D* = [*X*_*i*_,*i* = 1,2,⋯,*N*], *X_i_* was a 192 × 192 FC matrix, Subscript *i* refered to the subject, N represented the total number of subjects. In the identification step, the similarities between the target matrix and all the right hemisphere FC matrices in the dataset were calculated. These similarities were defined as Pearson correlation between the target matrix and each FC matrix in the dataset. When the target matrix (LH) and a matrix (RH) in the dataset obtained the maximum Pearson correlation value and their ID was the same [*ID* = *argmax*({*r*_1_,*r*_2_,⋯,*r*_*N*_})], it meant correct identification. The upper part of the dataset matrices in Figure 1B were the FC matrices of RH, that was, the contralateral hemisphere was used as a test set to identify individual. And the lower part of Figure 1B also contained all FC matrices of LH except the target matrix, namely using ipsilateral and contralateral hemisphere as a test set to identify individual. Similarly, the steps of the RH to recognize the LH were consistent with the above process. In order to evaluate the validity and robustness of this identification method in statistics, a non-parametric permutation test was performed. In each recognition process, we randomly shuffled the subjects’ hemispheres in the dataset, and then used each target matrix to identify them in turn, and compared the difference between the obtained recognition rate and the initial recognition rate. This process was performed 1,000 times. In order to explore the contribution of sub-modules to individual recognition, we carried out individual recognition for each of the five sub-modules, and the recognition steps were basically the same as the hemispheric recognition process. In the following content, we also defined the hemispheric similarity of each sub-module as HSFC.

### Age-related changes in hemispheric similarity

A large number of studies (29–32) had shown that aging could affect the FC between brain regions, not only the connectivity within functional subnetworks, but also the connectivity between different functional subnetworks. Aging caused the brain networks of older people to become less modular, as well as reduced local efficiency. In order to investigate the variation trend of HSFC during aging, we calculated Pearson correlation between subjects’ age and HSFC. At the same time, the above operations were also performed on five sub-modules.

### Validation analysis

In this study, individual identification and the relationship between HSFC and age were conducted based on AICHA template. In order to explore the stability of the calculation results for atlas, we used the human brainnetome atlas (BNA)^3^ for validation analysis (33). The BNA was based on a connective architecture that allowed brain anatomy to be correlated with psychological and cognitive functions and therefore was suitable for functional brain network analysis. The atlas divided the brain into 246 regions (123 for each hemisphere), comprising 210 cortical regions and 36 subcortical regions. It had been used in some studies to divide the brain for FC and brain network analysis (34–37). For each subject, referring to AICHA’s FC matrix construction process, finally we got a 246 × 246 symmetric FC matrix. Each intra-hemisphere was 123 × 123 symmetric FC matrix. The above AICHA’s results were repeated using the FC matrix obtained by the BNA. At the same time, we compared the robustness of HSFC between different templates. In addition, the above analysis was repeated with NGSR to explore the effect of global signal on the results.

## Results

### Hemispheric similarity of functional connectivity of hemispheres and sub-modules in different age group

**FIGURE 2:**
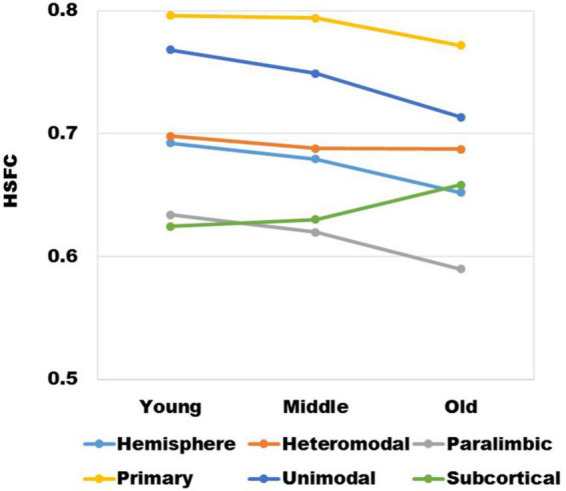
The variation trend of HSFC of hemisphere and each sub-module (heteromodal, parallel, primary, unimodal, subcortical) in the process of aging.

It could be seen from Figure 2 that with the increase of age, except for subcortical, HSFC in other sub-modules and hemispheric level showed a decreasing trend, and heteromodal had the smallest decline. For different age group, the HSFC of primary always maintained the maximum value, followed by unimodal and heteromodal. In youth and middle age, the HSFC of unimodal was higher than that of subcortical, while in old age, the HSFC of subcortical was slightly higher than that of unimodal. In addition, the HSFC of paralimbic was slightly larger than that of subcortical in youth. With the aging process, the HSFC of paralimbic continues to decline, and the gap between paralimbic and subcortical was growing.

### Individual recognition of hemispheric similarity of functional connectivity

**FIGURE 3:**
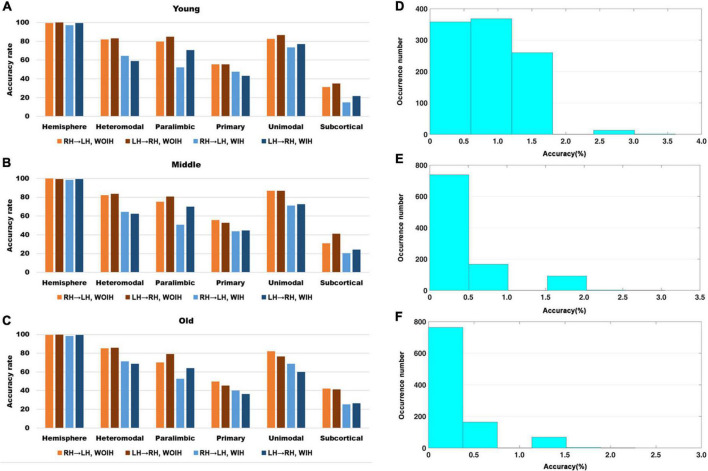
The recognition rate results of hemisphere and each sub-module in different age groups. **(A–C)** Represents the recognition rate of young, middle and old, respectively. From left to right in each sub graph, the recognition rate of hemisphere and five sub-modules are in turn. Notably, Orange indicates that the RH recognizes the LH without Ipsilateral Hemisphere (RH→LH, WOIH). Brown indicates that the LH recognizes the RH without ipsilateral hemisphere (LH→RH, WOIH). Light blue indicates that the RH recognizes the LH with ipsilateral hemisphere (RH→LH, WIH). Dark blue indicates that the LH recognizes the RH with ipsilateral hemisphere (LH→RH, WIH). **(D–F)** Correspond to non-parametric permutation test of **(A–C)**, respectively.

We first observed the individual recognition results without ipsilateral hemisphere from Figure 3. It could be found that the individual recognition results of different age groups were roughly the same. Among them, the recognition ability of hemispheric level was the best and that of subcortical was the lowest. Heteromodal, paralimbic and unimodal had similar recognition abilities, which were slightly higher than primary. After adding the ipsilateral hemisphere for recognition, the recognition ability of each sub-module decreased to varying degrees, while the hemisphere level recognition had little effect. In addition, for the difference of LH to recognize RH or RH to recognize LH, there was little difference at the hemispheric level, but there were partial differences in different sub-modules. Given that the identification trials were not independent from one another, we performed non-parametric permutation testing to assess the statistical significance of these results. Across 1,000 iterations, the highest success rates achieved were 6/166 (Young group), 6/197 (Midlle group),6/264 (Old group), neither of which exceeded 4%. Thus the *P*-value associated with obtaining at least correct identifications (the minimum rate we achieved) was 0.

### Age-related changes in hemispheric similarity

**FIGURE 4:**
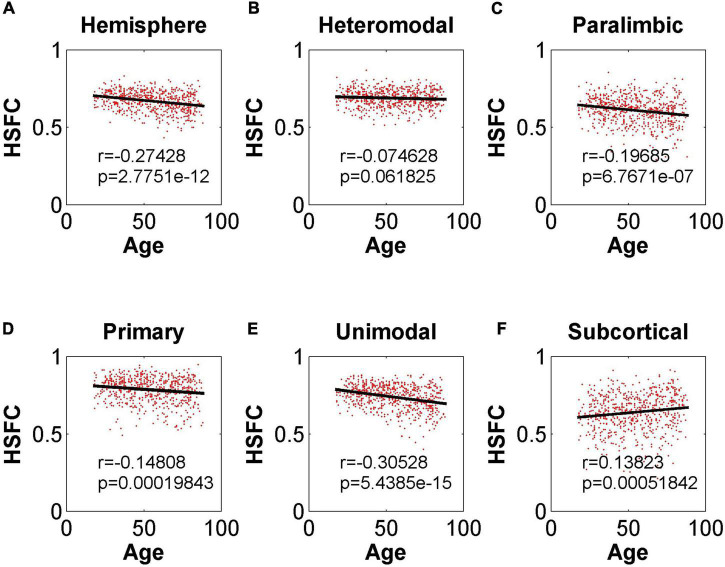
Pearson correlation between age and HSFC of hemisphere and five sub-modules. **(A–F)** Represent the hemisphere, heteromodal, paralimbic, primary, unimodal, subcortical, respectively.

As shown in Figure 4, except for the positive correlation between HSFC and age in subcortical, the hemispheric and other sub-modules reflected the negative correlation trend. In addition, except that the correlation between HSFC and age was not significant (*r* = –0.075, *p* = 0.06 > 0.05) in heteromodal, HSFC of other sub-modules and hemispheric showed a significant correlation with age to varying degrees.

### Validation analysis of template and processing method

**FIGURE 5:**
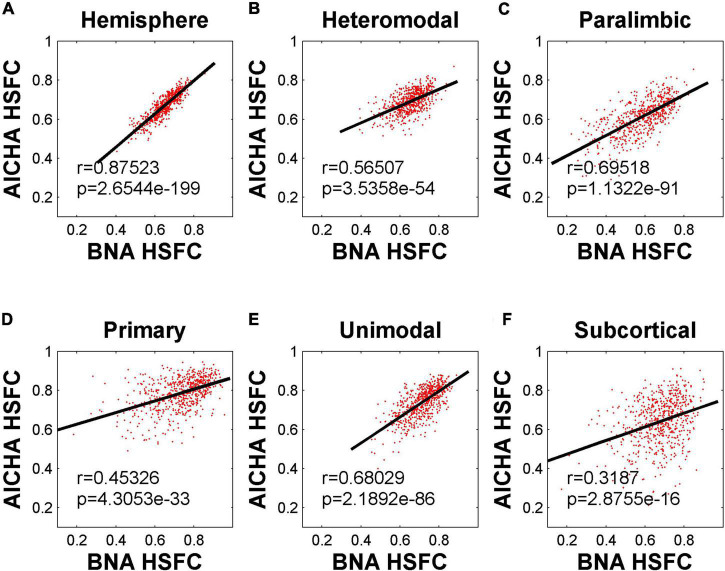
Pearson correlation of HSFC of hemisphere and five sub-modules between BNA and AICHA. **(A–F)** Represent the hemisphere, heteromodal, paralimbic, primary, unimodal, subcortical, respectively.

The HSFC used in the previous main work was based on AICHA template. In order to understand whether the HSFC was specific to AICHA template, we recalculated HSFC using BNA template, and then computed Pearson correlation on the HSFC obtained from the two templates. As shown in Figure 5, the HSFC between templates showed a very significant positive correlation (*r* = 0.88, *p* = 2.65 × 10^–199^) at the hemispheric level. The degree of correlation varied for different sub-modules. The correlation of subcortical was the lowest and the data points fitting were not strong.

**FIGURE 6:**
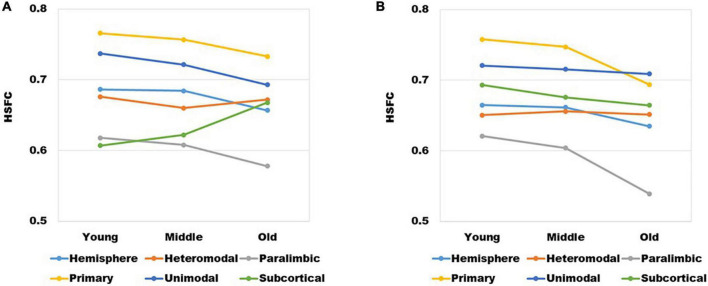
**(A)** The changes of HSFC with GSR obtained by BNA and **(B)** the changes of HSFC with NGSR obtained by AICHA in different age groups and modules.

The mean value of HSFC obtained by BNA was shown in Figure 6A. It seemed some differences when compared with the HSFC obtained by AICHA (Figure 2). Except for hemisphere and heteromodal, the mean value and change trend of other modules were basically similar to the HSFC obtained by AICHA. The HSFC with NGSR obtained by AICHA was shown in Figure 6B and the HSFC distribution was different from that obtained by GSR (Figure 2). Except for heteromodal, the mean value of HSFC in other modules decreased with aging. In addition, the value of subcortical was higher than that of GSR and was greater than that of heteromodal in different age groups, while the HSFC of unimodal was higher than that of primary in old age. However, the contrast of HSFC between hemisphere and heteromodal in youth and middle age was opposite to that with GSR.

**FIGURE 7:**
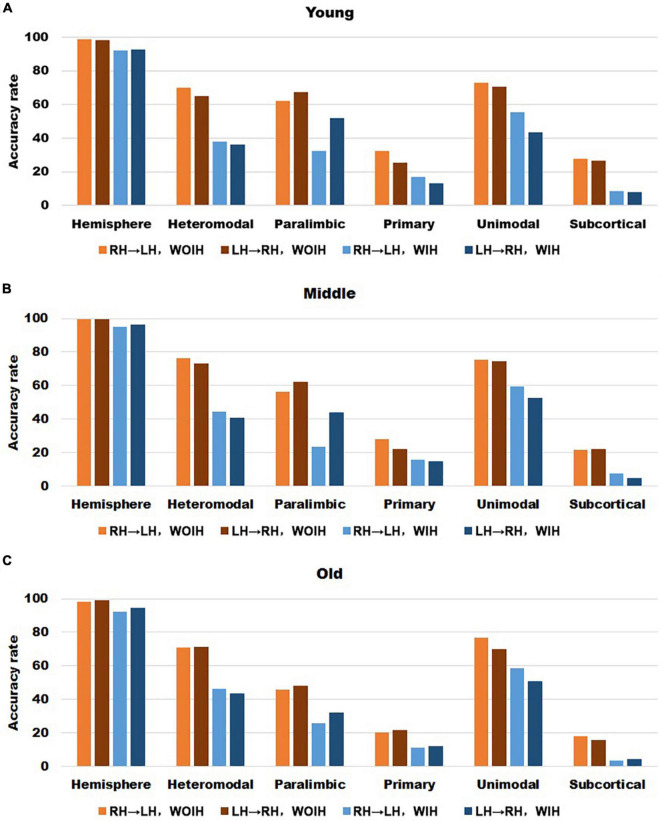
The recognition rate results of hemisphere and each sub-module in different age groups for BNA template with GSR. **(A–C)** Represents the recognition rate of young, middle and old, respectively. From left to right in each sub graph, the recognition rate of hemisphere and five sub-modules are in turn. Notably, orange indicates that the RH recognizes the LH Without Ipsilateral Hemisphere (RH→LH, WOIH). Brown indicates that the LH recognizes the RH Without Ipsilateral Hemisphere (LH→RH, WOIH). Light blue indicates that the RH recognizes the LH With Ipsilateral Hemisphere (RH→LH, WIH). Dark blue indicates that the LH recognizes the RH With Ipsilateral Hemisphere (LH→RH, WIH).

**FIGURE 8:**
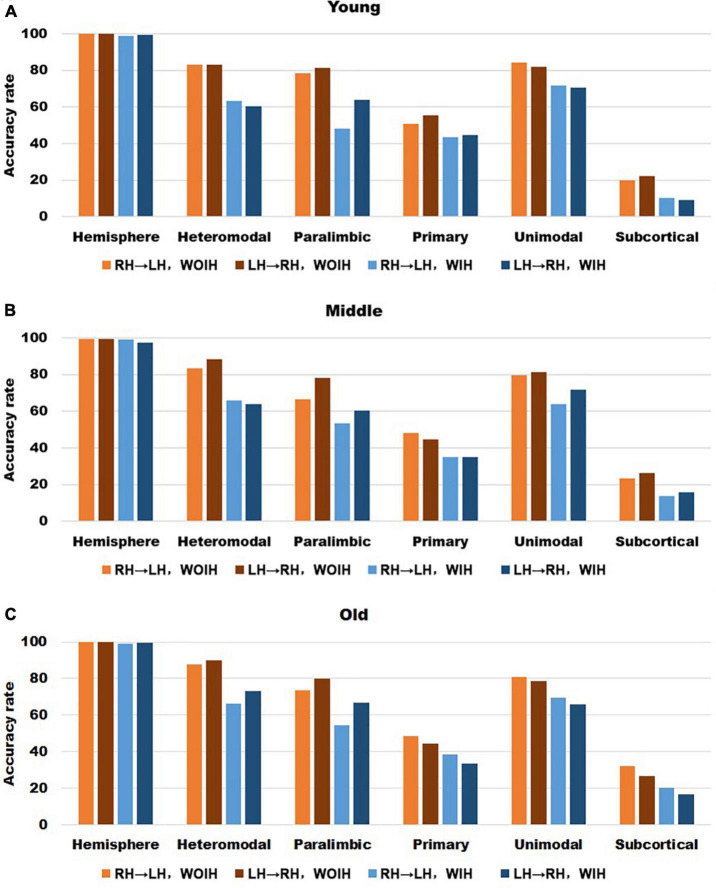
The recognition rate results of hemisphere and each sub-module in different age groups for AICHA template with NGSR. **(A–C)** Represents the recognition rate of young, middle and old, respectively. From left to right in each sub graph, the recognition rate of hemisphere and five sub-modules are in turn. Notably, orange indicates that the RH recognizes the LH Without Ipsilateral Hemisphere (RH→LH, WOIH). Brown indicates that the LH recognizes the RH Without Ipsilateral Hemisphere (LH→RH, WOIH). Light blue indicates that the RH recognizes the LH With Ipsilateral Hemisphere (RH→LH, WIH). Dark blue indicates that the LH recognizes the RH With Ipsilateral Hemisphere (LH→RH, WIH).

In the validation part of recognition rate, first of all, we observed the recognition rate results obtained from the BNA template (Figure 7). The individual recognition rates of paralimbic, primary and subcortical in the elderly were lower than those in the youth and middle age. In the comparison of the recognition rate of different templates, it was found that the recognition rate of AICHA template was better than that of BNA template, especially at the sub-module level. Next, after comparing the recognition rate results of GSR (Figure 3) and NGSR (Figure 8), we could find that the recognition rate of each module was almost the same.

**FIGURE 9:**
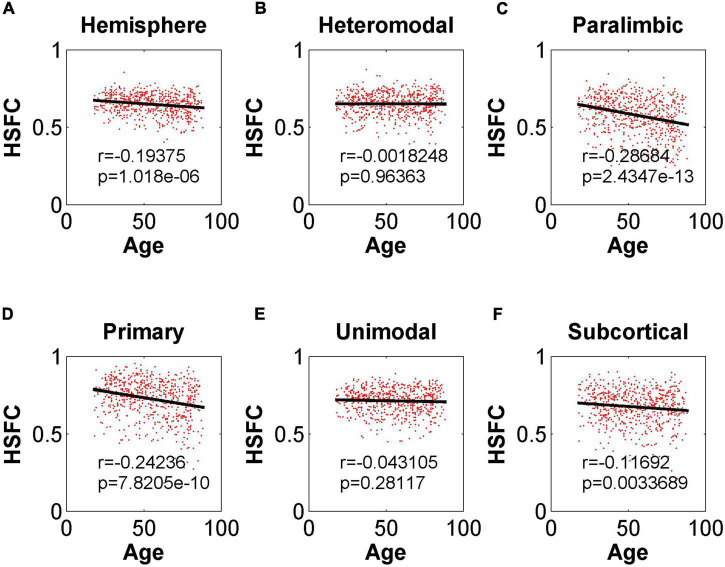
Pearson correlation between age and HSFC of hemisphere and five sub-modules for BNA template with GSR. **(A–F)** Represent the hemisphere, heteromodal, paralimbic, primary, unimodal, subcortical, respectively.

**FIGURE 10:**
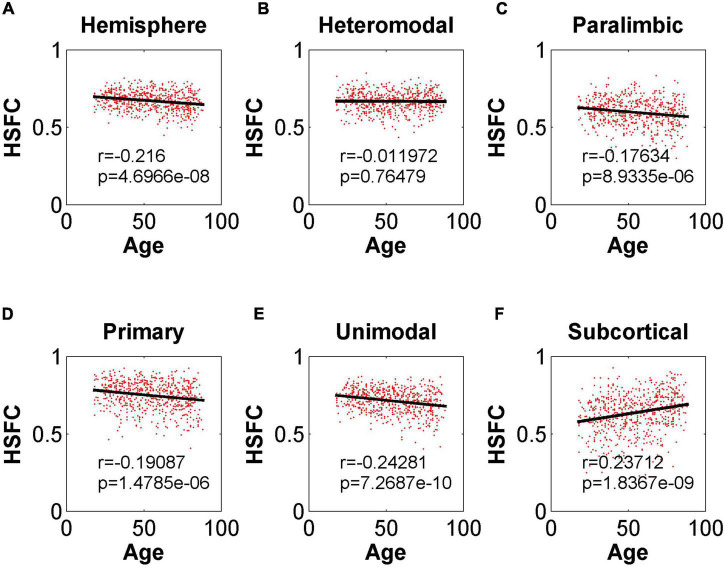
Pearson correlation between age and HSFC of hemisphere and five sub-modules for AICHA template with NGSR. **(A–F)** Represent the hemisphere, heteromodal, paralimbic, primary, unimodal, subcortical, respectively.

In the validation part of the correlation between HSFC and age, similarly, we used the BNA template for validation (Figure 9) and found that the distribution patterns between the two templates were similar. The Pearson correlation between the HSFC and age for heteromodal and unimodal had no significant difference (heteromodal: *r* = –0.002, *p* = 0.96; unimodal: *r* = –0.043, *p* = 0.28). The correlation coefficient obtained by primary and paralimbic was larger than that of the corresponding sub-module in AICHA template. In addition, the correlation value between HSFC and age of primary and subcortical with NGSR (Figure 10) was higher than that with GSR, and the other modules were the opposite.

## Discussion

In this study, the Cam-CAN datasets with a large age span were used and the index named “hemispheric similarity of functional connectivity (HSFC)” was proposed. This index could effectively identify individuals and reflect the change trend in the aging process. In addition, the results obtained in different sub-modules were also different. The results were robust to different templates and whether the global signal was regressed or not. This proves that HSFC has unique advantages in aging research, and also shows that HSFC has the characteristics of individual differences.

The specificity of the cerebral hemisphere is a sign of successful neural development (38). Previous studies (39–41) extracted a series of indicators as features through the specificity of hemispheric function or the asymmetry of hemispheric structure and function, and obtained a high accuracy in the diagnosis of diseases. On the contrary, some studies (42, 43) found the conclusion of hemispheric asymmetry through the processing and statistical analysis of imaging data. The above studies indirectly revealed the importance of the cerebral hemisphere, suggesting the starting point of this study.

In the past, the application of pattern recognition in imaging research was generally by extracting the features of different levels of the brain and building a classifier for the prediction of category variables or building a regressor for the continuous variables of behavior scores. Next, using a new test set to get the results on the classifier or regressor. Different from the common pattern recognition methods, this study based on the “fingerprint” method proposed by Finn et al. (9) had achieved a very high accuracy at the hemispheric level, which showed that each individual is unique. For sub-modules, since the primary module mainly involves primary cortical areas such as the central gyrus (25), the FC similarity of homologous brain areas between hemispheres is also very high and the degree of lateralization is small. Therefore, its HSFC value was the highest among all modules (Figure 2). Meanwhile the functional patterns of the primary module in the LH and RH are very similar so that the individual differences at the group level are not high, which led to a low recognition rate (Figure 3). Subcortical module mainly involves subcutaneous nuclei (25). The segmentation effect of subcutaneous nuclei in image data preprocessing is poor, which also indirectly affects the calculation of HSFC, resulting in its generally low value. Therefore, the individual recognition ability was not strong. For the difference of recognition rate in sub-modules, we hypothesized that this might be due to differences in functional connectivity similarities between homologous brain regions of different modules, leading to differences in the degree of lateralization, and thus affecting HSFC. We believe that HSFC can better reflect the degree of lateralization in different brain regions. The higher the value of HSFC, the higher the similarity of functional connectivity of homologous brain regions in this region, and the smaller the degree of lateralization. The smaller the value of HSFC is, the lower the similarity of functional connections of homologous brain regions in this region, and the greater the degree of lateralization. This can help us further explore differences in the degree of lateralization in different regions of the brain. In different age groups, the results of recognition rate were basically the same, which also showed that the individual differences of HSFC were stable in the aging process and had good robustness.

In the previous study (13), it was found that the changes of vascular components, head movements and the location of functional areas would affect the relevant patterns of FC and aging process, so a series of analysis and processing methods were proposed. Another study (44) showed that the shrinkage rate of various regions of the cerebral cortex during aging was not the same. In this study, based on the relationship between the HSFC and age, we found that HSFC decreased with the aging process on the whole. The results showed that the aging process led to the pattern disorder of many functional subnetworks, which disrupted the symmetry of hemispheric functional networks to some extent and further provided valuable clues for the future study of the development pattern of hemispheric functional networks in the aging process.

Through the study of the HSFC between different templates, it showed that HSFC is not only specific to a fixed template, but also could be extended to more functional templates. When using BNA template or NGSR, the results obtained were basically consistent with our main results (i.e., GSR with AICHA template).

In the outlook of the follow-up work, first of all, the FC network of this study was calculated by Pearson correlation. Some studies (45, 46) proposed the processing strategy of “distance correlation” and its research results were better than Pearson correlation, which was worthy of our reference in the future. Second, although this study used a wide range of aging data, it was limited to rs-fMRI research. In the future, structural MRI and task fMRI can be added for a more comprehensive analysis or we can consider applying the HSFC-based method to HCP datasets with different scans, so as to verify the recognition stability of HSFC at different time points in the same individual. Third, this study was aimed at a series of conclusions obtained in the process of normal aging, and its application prospect in Alzheimer’s disease and other nervous system and mental diseases is not clear. Fourth, the continuous optimization of preprocessing strategy and the realization of large sample data are still big problems that have been committed to research in the field of pattern recognition, which still need to be solved.

In this study, the HSFC was proposed for the first time and it could effectively identify individuals and reflected the changes in the aging process. In particular, we found that there are differences in the recognition rate among sub-modules and there were also differences in the trend with age. Finally, as a validation, we repeated the main results through another functional template and NGSR, which had good robustness. This also provides new clues for the pattern of changes between hemispheres in the normal aging process.

## Data availability statement

Publicly available datasets were analyzed in this study. This data can be found here: http://www.cam-can.org/.

## Ethics statement

The studies involving human participants were reviewed and approved by the University of Cambridge’s Research Ethics Committee. The patients/participants provided their written informed consent to participate in this study.

## Author contributions

The author confirms being the sole contributor of this work and has approved it for publication.

## Footnotes

1
http://www.cam-can.org/

2
https://fsl.fmrib.ox.ac.uk/fsl/fslwiki/FUGUE

3
http://atlas.brainnetome.org/
