# Corrigendum: Individual prediction of hemispheric similarity of functional connectivity during normal aging

**DOI:** 10.3389/fpsyt.2022.1035694

**Published:** 2022-10-19

**Authors:** Yingteng Zhang

**Affiliations:** Department of Mathematics, Taizhou University, Jiangsu Province, Taizhou, China

**Keywords:** hemispheric similarity of functional connectivity, functional MRI, normal aging, individual recognition, global signal

In the published article, there was an error in the affiliation of “Yingteng Zhang”. Instead of “Department of Mathematics, Taizhou University, Taizhou, China,” it should be “Department of Mathematics, Taizhou University, Jiangsu Province, Taizhou, China”.

The authors apologize for this error and state that this does not change the scientific conclusions of the article in any way. The original article has been updated.

## Publisher's note

All claims expressed in this article are solely those of the authors and do not necessarily represent those of their affiliated organizations, or those of the publisher, the editors and the reviewers. Any product that may be evaluated in this article, or claim that may be made by its manufacturer, is not guaranteed or endorsed by the publisher.

